# Suggested Irish Board to Grant Diplomas to Nurses

**Published:** 1917-03-10

**Authors:** 


### THE NURSING PROBLEM IN IRELAND.

### Suggested Irish Board to Grant Diplomas to Nurses.

A remarkable and interesting development of the nursing situation is taking place in Ireland. The Colleges of Physicians and Surgeons, having appointed a Committee to examine the present situation arising from the proposals of the Royal British College of Nursing, a recommendation has been made by this Committee that an Irish Board should be formed having power to grant diplomas to nurses. At present the scheme is *in nubibus*, the deliberations of the Committee and the Colleges not having been completed. But in all probability it will mature, and an Irish Nurses' Board, dominated by the Irish Medical Faculty, will result. The object is probably twofold—to side-track the R.B.C.N. movement in Ireland, and to secure State Registration independently. Should the Royal British College of Nursing at any time approach Parliament to claim State Registration for Nurses whose names are on its register, Irish nurses will at the same time come forward with a similar claim, and, backed by the Conjoint Board as regards qualifications, they consider that their demand will be irresistible. This movement is distinctly strong, and in all probability will have the effect, at least temporarily, of paralysing the efforts of the Irish Board formed by the R.B.C.N. Ultimately there may even be a union of forces. The situation is unique, and in general terms is as follows. Some of the principal matrons in Ireland, altogether a score or thereabouts, are divided into two camps, and one of them has secured the support of the Irish medical profession. The other, favouring the R.B.C.N., is regarded as "the English," although as a matter of fact there are quite as many English, or English trained, on one side as on the other. The parties are probably divided more by temperament than by questions involving principle. Both are in pursuit of registration and of raising the status of the nursing profession. And the peculiar feature of the situation is that no effort has been made to discover the wishes, if any, of the rank and file. No referendum has been taken, no public meetings held, so that for recruiting purposes the field is open.

### Registration.

In my opinion much profit might ensue from a frank discussion of the subject of registration. That it is very desirable of attainment is probably admitted by all, but there are many who regard registration as a panacea for all the ills of the profession. I am of those who do not, and who on this account regard the potentialities of the R.B.C.N. as superior to those of any party or parties who have made registration their one and only aim. The reasons upon which I base this view may perhaps merit consideration. Registration in a profession stands for what a trade union means to a trade. It helps to confine practice of the profession or trade to a limited number, that number consisting of persons who have undergone a specified course of training or preparation. The value of registration in every case depends on the success with which this purpose is or can be achieved. There are certain professions and trades in which success is for all practical purposes complete. The Bar and the solicitors' profession are probably the most conspicuously successful; medicine and pharmacy probably come next. In dentistry registration is by no means a complete success, inasmuch as there are far more unregistered than registered dentists in the United Kingdom, some of whom have large and lucrative practices in the West End of London. In recent years the teaching profession obtained a register, and this is a case in which the value of a charter is relatively small. The explanation is that it will never be possible to limit the teaching profession to qualified persons, no matter how many standards are set up. The engineering profession is without a register, and probably because its leaders appreciate that limitations to practising are impossible to establish. The Hon. Charles Parsons is accredited with the statement that any travelling tinker may with impunity add the letters C.E. after his name. Any ignorant adventurer, similarly, may open a seminary for the sons of gentlemen.

### A Simple Issue.

Is it possible to limit the nursing profession by means of a register? I put this question to all whom it may concern, and I submit that, save in midwifery, it is impossible. No Legislature, for example, could or would attempt to prevent a mother from nursing the members of her family, and the same applies to a sister, a brother, or a friend. The term "nursing" is at least as indefinite in its scope as "teaching" or "engineering." Much, obviously, depends on the truth of this proposition. If it can be established that registration is potential to limit the number of nurses in the country, or, in other words, to penalise general nursing by unlicensed persons, then the registrationists' claim is proven. The subject becomes of supreme importance, and, comparatively, nothing else matters. But, on the other hand, if it can have no such effect, if registration would merely standardise a certain number, and leave the field open, as is at present the case in the teaching profession, then the case of the R.B.C.N. is overwhelmingly powerful. The College, it appears to me, proposes to take under its wing all persons engaged in nursing, to classify them in such a way as to prevent confusion, just as the crew of a battleship are classified. The men are all sailors, yet there is not the smallest chance of an admiral being mistaken for a captain or a captain for a boatswain.

### The V.A.D.s.

There is much bother about the V.A.D.s. I do not propose to waste words discussing the patriotism, or the good qualities, or the absolute necessity for these ladies. For the purposes of this argument these things do not matter. The fact of importance that remains is that the V.A.D.s are with us, and when the war is over they will be with us in greater force than now. This will happen whether there is or is not legislation, whether the College grows or dies. In the opinion of any reader of this letter, if these ladies desire to engage in nursing for remuneration, privately or in a hospital, is it likely that they will be prohibited from so doing by law in the event of State Registration becoming a *fait accompli*? In the opinion of the writer this is not remotely probable. They will, I am convinced, be quite as free as they are now, and therefore the proposal of the College to organise and certificate them according to their value appears to me to be an admirably conceived and businesslike proposition.

### Representation.

Democratic control is unfortunately lacking in nursing circles. There is as little of this spirit apparent in our Irish Nurses' Association as its leading spirits attribute to the College. Is it not possible for the matron of broad mind to advocate an independent and thinking spirit amongst the members of her staff? In Ireland, at the present moment, where a matron attaches herself to one or other of the two great causes, it is usual to tick off the hospital whose nursing staff she controls as though the entire staff had expressed an opinion, and as if the matron carried all their proxies. The experienced sister, the independent private nurse, the municipal nurse—these are never heard of in great movements, and active preparations are even now being made to legislate for them without their consent. The time has come to make the issues plain to every member of the profession, whether of high rank or humble—to enter vigorously on a campaign in which the younger members will actively engage, and in the issues of which they will feel that they have a direct interest. The air requires to be cleared of all that appertains to narrowness or prejudice, and these appear to gather round registration like clouds round a mountain-top. That which in itself is intrinsically good is not necessarily the *summum bonum*, nor a remedy for every disease under the sun.

IERNE.

